# γ-Synuclein as a marker of retinal ganglion cells

**Published:** 2008-08-22

**Authors:** Irina Surgucheva, Alejandra D. Weisman, Jeffrey L. Goldberg, Alexander Shnyra, Andrei Surguchov

**Affiliations:** 1 Retinal Biology Research Laboratory, Veterans Administration Medical Center, Kansas City, MO; 2 Department of Neurology, Kansas University Medical Center, Kansas City, KS; 3 Bascom Palmer Eye Institute, University of Miami, Miami, FL; 4 Department of Pharmacology and Microbiology, Kansas City University of Medicine and Biosciences, Kansas City, MO

## Abstract

**Purpose:**

Previous studies have described γ-synuclein as a protein highly expressed in retinal ganglion cells (RGCs), and a loss of RGCs correlates with a downregulation of γ-synuclein gene expression in glaucoma. Here we asked whether γ-synuclein expression in the retina can be considered a specific marker of RGCs.

**Methods:**

γ-Synuclein expression was examined with immunohistochemistry in retinal sections from normal and glaucomatous human eyes. Primary cultures of RGCs from Sprague-Dawley rats purified by sequential immunopanning using a monoclonal antibody to Thy1–1, cultures of A7 immortalized optic nerve astrocytes from newborn rats, and the immortalized RGC-5 cell line were studied using immunofluorescence and quantitative RT–PCR.

**Results:**

γ-Synuclein was highly expressed in RGCs in the human retina and was localized in cytoplasm adjacent to the RGC nuclear marker, Brn-3a. Axons of RGCs were immunopositive for γ-synuclein in the nerve fiber layer (NFL), the lamina cribrosa and the retrobulbar optic nerve. In the optic nerve of glaucoma patients, axon swellings were likewise immunopositive, whereas in the retina of patients with retinoblastoma, NFL staining appeared reduced. In primary rat RGCs and in immortalized RGC-5 cultures, γ-synuclein was localized predominantly in the perinuclear area and in cell processes. Among rat retinal cells in culture, all Brn-3a positive cells were stained with a γ-synuclein antibody; rare γ-synuclein-positive cells were not stained by the Brn-3a antibody.

**Conclusions:**

γ-Synuclein is selectively and abundantly expressed in human RGCs in vivo, primary rat RGCs in vitro, and immortalized RGC-5 cells. In pathology, γ-synuclein abundance may vary between RGC somas and axons. Coincident Brn-3a and γ-synuclein expression suggests that strong γ-synuclein expression can be considered a marker of RGCs. Future translational approaches might include using a γ-synuclein promoter for the specific delivery of siRNA or therapeutic proteins to RGCs.

## Introduction

Glaucoma results in a slow, progressive, and selective dysfunction and ultimately apoptotic death of retinal ganglion cells (RGCs), the retinal neurons that project to the brain via the optic nerve [1-6]. The precise mechanisms involved in glaucomatous RGC death are not completely understood, but it is widely accepted that pathophysiological events in the retinal ganglion cell layer (GCL) and at the optic nerve head, through which RGC axons pass, play a prominent role in the development of this neuropathy. Therefore, a better appreciation of the factors involved in ganglion cell death is central to the development of therapeutic strategies [1,2]. RGCs make up only approximately 45% of RGC layer neurons in the mouse [7]. RGCs can not be reliably distinguished from the other neurons in the GCL, such as displaced amacrine cells, based on cell morphology alone [7,8]. In addition, RGCs are structurally and functionally heterogeneous, and information regarding the molecular composition and characteristics of RGC subtypes remains incomplete. Further progress in this field is difficult because of the paucity of markers distinguishing RGCs and their subtypes.

Finding and characterizing markers for RGC death could be an important step in understanding the molecular and cellular processes in glaucoma. Thy1–1 and Brn-3 are currently used as markers of RGCs, but Thy1–1 is not a good marker of RGC loss in models of retinal damage [9], and Brn-3c is only expressed in 50% of RGCs [10-12].

Here we show that γ-synuclein is highly and specifically expressed in RGCs in the intact retina, in primary cultures of RGCs, and in an immortalized cell line, RGC-5. We further demonstrate that γ-synuclein localization changes in the glaucomatous retina, and may thus be used as a marker for damaged RGCs in glaucoma.

## Methods

### Retina and optic nerve: tissue section preparation and staining

Human eyes, from donors aged 53–70 years who had no history of eye disease, were obtained from Mid-America Transplant Services (St. Louis, MO). Time from death to preservation was 4–8 h.

Retinas from two different one-year-old patients’ eyes, each enucleated for retinoblastoma, were compared with the retina of a control individual of the same age that died as a result of an accident. Paraffin-embedded blocks were received from Dr. Morton Smith (Department of Pathology, University of Wisconsin, Madison, WI). The preparation of samples was conducted in accordance with the tenets of the Declaration of Helsinki and approved by the Institutional Review Board of the University of Wisconsin. Samples of optic nerve were received from Dr. Martin B. Wax (Research and Development, Alcon Research Ltd, Fort Worth, TX). The donating patients were 61–72 years old at the time of death and had primary open-angle glaucoma. Clinical findings of these patients were documented for a follow-up period of five to eight years. Neither these donors nor the control 70-year-old individual were reported to have diabetes, collagen vascular disease, or sepsis. The cause of death for these donors was acute myocardial infarction or cardiopulmonary failure. Other characteristics of these patients are described in [13]. The eyes were enucleated within 4 h after death and processed and fixed within 6 h in either 10% buffered formaldehyde or 4% paraformaldehyde. The posterior poles were dissected free of surrounding tissues, washed extensively in 0.2% glycine in phosphate-buffered saline (PBS; 0.8% NaCl, 0.02% KCl, 0.144% Na_2_HPO_4_, 0.024% KH_2_PO_4_, pH 7.4) and embedded in paraffin. The sections were prepared as described earlier [13].

### Immunofluorescent staining

Human retinas from donors without eye diseases were stained with a 1:1000 dilution of monoclonal γ-synuclein antibody (clone 1H10D2; Antagene, Mountain View, CA) and 1:2000 antirabbit polyclonal Brn-3a antibody (gift of Dr. Eric E. Turner, University of California San Diego, La Jolla, CA), followed by a 1:1000 dilution of fluorescent secondary antibodies antirabbit-Alexa-568 and antimouse-Alexa-488 (Invitrogen, Carlsbad, CA).

### Immunohistochemical staining of retinas from retinoblastoma patients

After enucleation, eyes were washed in PBS containing 0.1% glycine and processed for paraffin embedding. Next, 8–10 5 µm slices were cut and applied to silane-coated slides (Fisher brand Super frost Plus slides catalog number 12–550–15; Fisher Scientific, Pittsburgh, PA). Before immunostaining, the sections were deparaffinized and incubated for 1 h in PBS-glycine (0.01% glycine in PBS, pH 7.4; Sigma-Aldrich, St. Louis, MO) at room temperature to reduce nonspecific binding. Slides were further preincubated with 5% milk for 30 min, and then rinsed and incubated with primary antibodies (1:100) against γ-synuclein (Abcam, Cambridge, MA) for 30 min. A biotinylated rabbit secondary antibody (Vectastain Elite ABC kit; Vector Laboratories, Burlingame, CA) was placed on the sections and incubated for 30 min, washed off with PBS, and exposed to streptavidin-peroxidase conjugate (Vector Laboratories) for 30 min. The bound antibody-peroxidase complexes on the sections were visualized using a 3, 3-diaminobenzidine tetrahydrochloride (DAB) substrate solution consisting of 1.5 mg DAB and 50 μl of 30% hydrogen peroxide in 10 ml of 0.1 M Tris-HCl, pH 7.6. The sections were incubated in the dark until brown staining appeared, washed in PBS, counterstained with hematoxylin, dehydrated, and coverslipped with Permount. Control sections were run in parallel, omitting only the primary antibody.

### Preparation of rat retinal cells

After dissection from postnatal day 0 Sprague-Dawley rats (Charles River Laboratories, Wilmington, MA), retinas were digested with papain (165u/10 ml; Worthington Biochemical, Lakewood, NJ) for 30 min and then triturated mechanically until all the cells were in suspension. The cells were seeded on coverslips precoated with poly-D-lysine and laminin at a density of 50,000 cells per coverslip. The cells were incubated overnight at 37 °C with 10% CO_2_ in serum-free Neurobasal media (Invitrogen) containing 5 µg/ml insulin, 50 µg/ml brain-derived neurotrophic factor (BDNF), 10 µg/ml ciliary neurotrophic factor (CNTF), and 10 µg/ml forskolin.

### Primary cultures of retinal ganglion cells

For primary cultures of RGCs from Sprague-Dawley rats, the cells were purified by sequential immunopanning to a 99.5% purity [14] using a monoclonal antibody to Thy1–1 as described [14-17].

Briefly, whole retina tissue was removed from rats from four litters, incubated in a papain solution as described above, dissociated into single-cell suspension, and incubated with 1:100 antimacrophage antiserum (Axell Accurate Chemical and Scientific Corp, Westbury, NY). The retinal cell suspension was passed over panning plates coated with goat anti-rabbit secondary antibodies to remove macrophages and endothelial cells. The remaining, unbound cells were then placed on panning plates containing Thy1–1 antibody (T11D7) to bind RGCs, and unbound cells were rinsed away. RGCs were released after incubation with a trypsin solution. RGCs were cultured overnight on poly-D-lysine (Sigma-Aldrich) and 2 mg/ml laminin (Telios/Invitrogen, Carlsbad, CA) in serum-free defined medium containing 50 ng/ml BDNF, 10 ng/ml CNTF, 5 µg/ml insulin, and 5 µM forskolin as described [14,16]. These cultures typically generate a yield of RGCs between 50% and 70%, with a survival of more than 80% over the first day in culture [14].

### A7 astrocytes from rat optic nerve

The culture of immortalized optic nerve astrocytes from newborn rats was a generous gift of Dr. Herbert Geller (NHLBI, NIH, Bethesda, MD). The cells were grown as described previously [18] in DMEM with 2 mM glutamine, 10% FBS, 0.45% glucose in the presence of penicillin/streptomycin (final concentration of penicillin 100 IU/ml, streptomycin 100 μg/ml). The cells expressed an astrocyte-specific marker, glial fibrillary acidic protein (GFAP).

### Culture of the rat retinal ganglion cell line, RGC-5

RGC-5 cells [19] were kindly provided by Dr. Raghu Krishnamoorthy, Department of Cell Biology and Genetics, University of North Texas Health Science Center, Fort Worth, TX. The cells were maintained in Dulbecco’s modified Eagle’s medium containing 10% fetal bovine serum (BRL, Gaithersburg, MD), 100 U/ml penicillin, and 100 µg/ml streptomycin (Sigma-Aldrich). The cells were grown in a humidified atmosphere of 95% air and 5% CO_2_ at 37 °C. The RGC-5 cells have a doubling time of approximately 18–20 h and were passaged by trypsinization every 3–4 days as previously described [20].

### Immunofluorescent staining of RGC-5 cells

**Figure 1:**
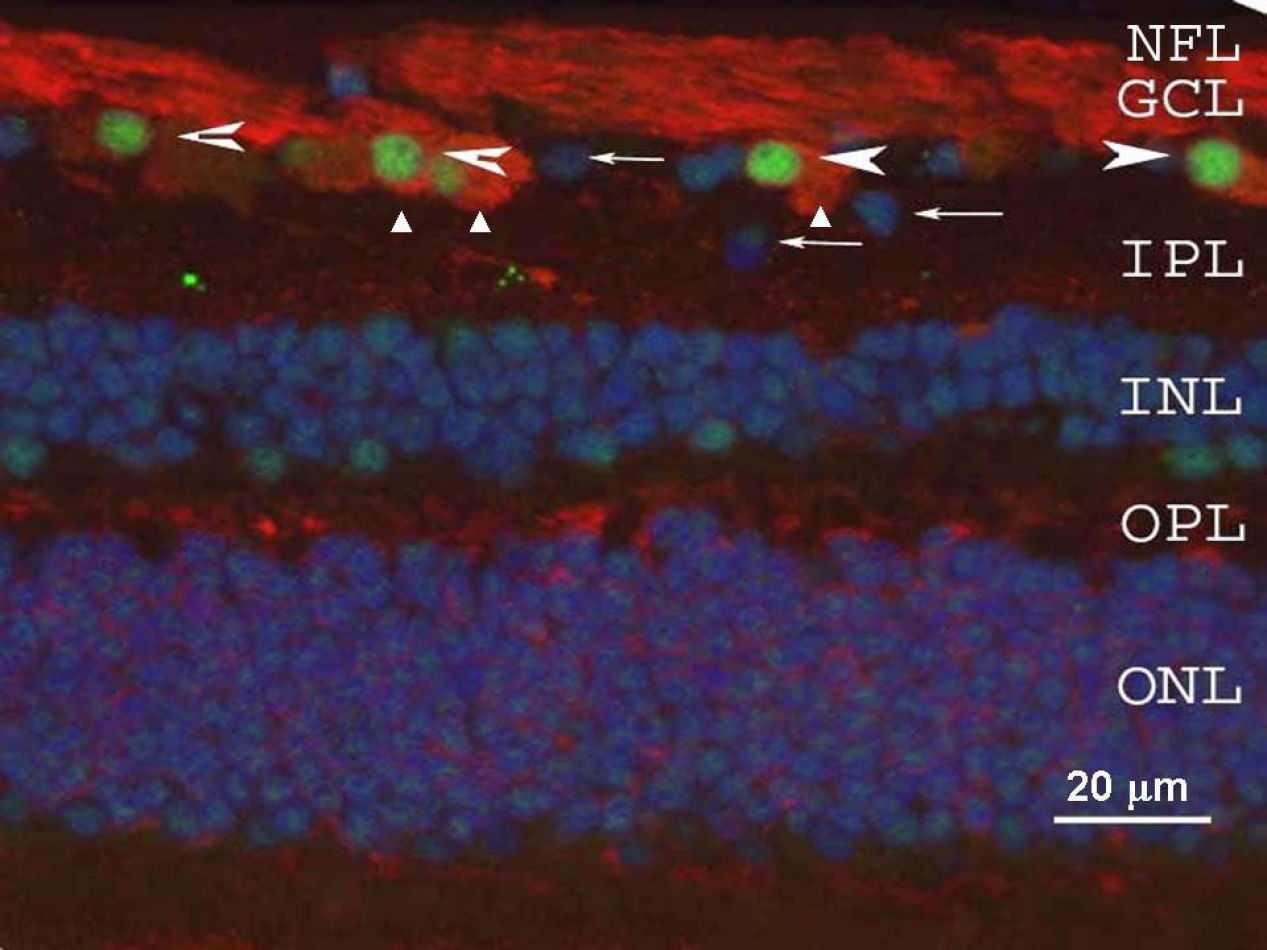
γ-Synuclein and Brn-3a localization were examined in the human retina by immunofluorescence. γ-Synuclein (red) was present in the cytoplasm of retinal ganglion cells (RGCs; triangles) and in the nerve fiber layer (NFL), while Brn-3a (green, arrowheads) was observed in RGC nuclei. Arrows mark cells in the retinal ganglion cell layer (GCL) that were not stained by either Brn-3a or γ-synuclein. Nuclei throughout the retina are counterstained with DAPI (blue), and little immunofluorescence is noted through the inner or outer plexiform layers (IPL and OPL, respectively), or inner and outer nuclear layers (INL and ONL, respectively). Thus γ-synuclein is localized both in the body of RGC and in their axons.

Immunofluorescent staining of an immortalized cell culture (RGC-5) was done as described previously [21] with slight modifications. Briefly, cells were split on glass coverslips treated with 0.02% poly-D-lysine. Before staining, the cells were washed with PBS, fixed with 100% methanol for 10 min at –20 °C, and then fixed with a 1:1 methanol: acetone mixture for 4 min at −20 °C. After washing by PBS (4 times, 10 min each washing), the coverslips were treated with 5% BSA in PBS-T (PBS + 0.3% Triton X-100) for blocking unspecific binding (1 h, room temperature). The coverslips were incubated overnight at 4 °C with a 1:150 dilution γ-synuclein polyclonal antibody (Abcam, Cambridge, MA). After washing, the coverslips were incubated with a 1:300 dilution of secondary antirabbit antibodies to which Cy2 was conjugated (Jackson Laboratories, Bar Harbor, ME). After another washing, the coverslips were loaded on mounting solution containing DAPI (Invitrogen), and fluorescent images were recorded with a Leica DMI 6000B inverted microscope.

### Immunostaining of retinal cell suspension and primary cultures of retinal ganglion cells

**Figure 2:**
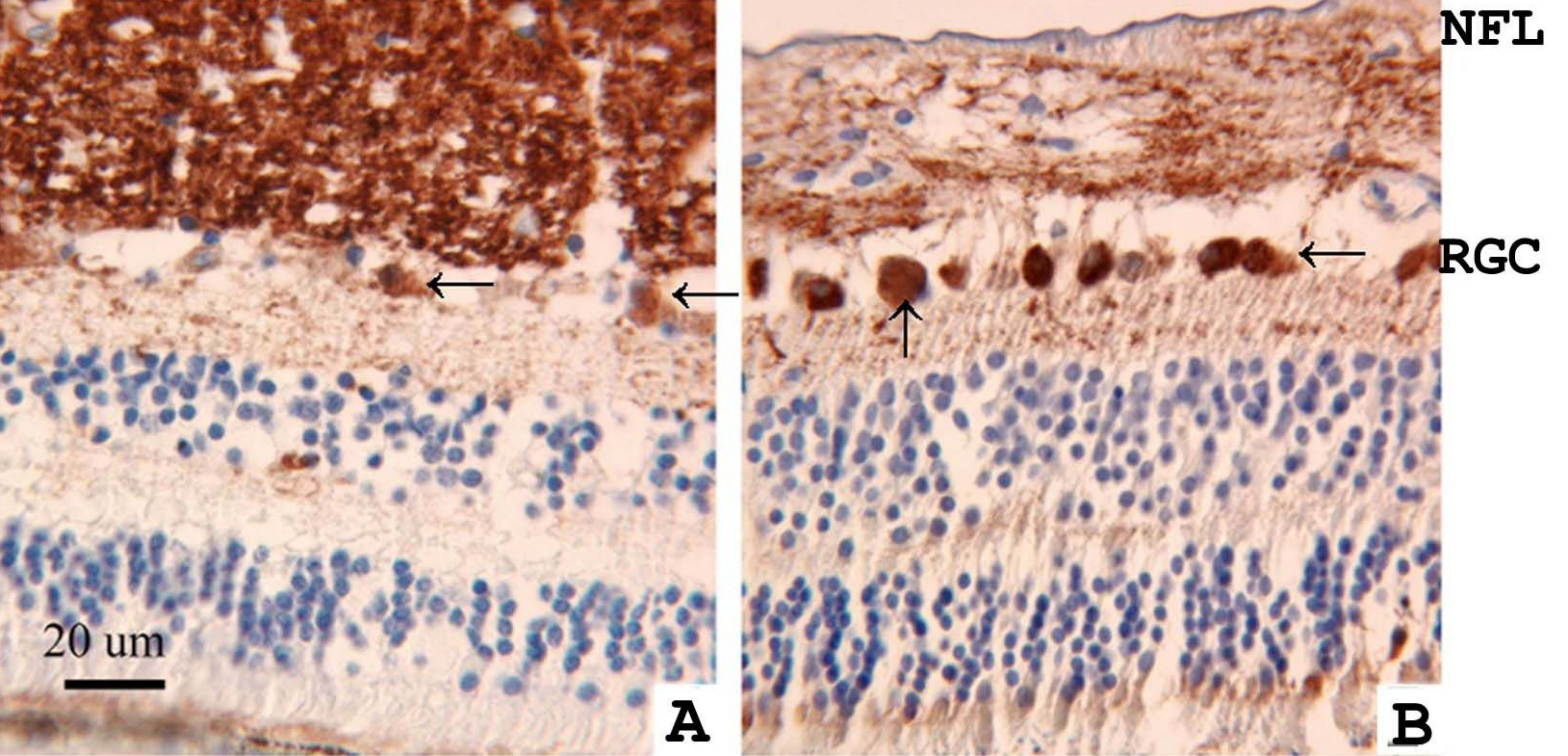
Localization of γ-synuclein was studied by immunohistochemistry in a normal human retina (**A**) and in the retina of an age-matched patient with retinoblastoma (**B**). γ-Synuclein was detected using a diaminobenzidine reagent (brown, peroxidase). Tissues were counterstained with hematoxylin (blue). Sections were stained with an antibody that recognized γ-synuclein, but not the other members of the synuclein family. The retina of the retinoblastoma patient (**B**) demonstrated more intense γ-synuclein immunoreactivity in retinal ganglion cell bodies (arrows) and less intensive staining in the nerve fiber layer (NFL) compared with the normal retina (**A**).

Following overnight incubation, the staining of the rat primary culture was conducted as described in the section above for RGC-5 cells but with slight modifications. The coverslips were incubated with a 1:100 dilution of the γ-synuclein polyclonal rabbit antibody (Abcam) and a 1:100 dilution of a mouse monoclonal Brn-3a antibody (MAB1585; Chemicon, Temecula, CA) overnight at 4 °C. The next day the coverslips were washed with PBS (three times, 15 min each wash) on a shaker at room temperature and incubated with secondary antibodies for 90 min. As a secondary antibody we used highly cross adsorbed AlexaFluor488 goat antirabbit IgG (H^+^L) and AlexaFluor568 donkey antimouse IgG (Invitrogen). After washing, the coverslips were mounted in Vectashield with DAPI (Vector Laboratories Inc., Burlingame, CA), and fluorescent images were obtained with a fluorescence microscope (Axiovert 200M, Carl Zeiss, Germany).

### RNA isolation

RNA was isolated from 4x10^6^ cultured RGC-5 cells using the spin column method of an RNeasy Protect Mini Kit (Qiagen, Valencia, CA), and the RNA was stored at −80 °C. The isolated RNA was quantified by spectrophotometric absorbance assuming that 1 absorbance unit at 260 nm in 10 mM Tris-HCl, pH 7.5 corresponds to an RNA concentration of 44 µg/ml. Only samples with an A_260_/A_280_ ratio higher than 1.9 were used. The integrity of RNA was checked by denaturing 1.2% agarose gel electrophoresis in the presence of formaldehyde. rRNAs appeared as sharp bands, and the ratio of 28S rRNA to 18S rRNA quantified under UV light with Kodak Image Station 440CF (Eastman Kodak Co., Rochester, NY) was 2:1. The RNA was converted into cDNA using the High-Capacity cDNA Reverse Transcription Kit with random primers (Applied Biosystems, Foster City, CA).

### qRT–PCR

**Figure 3:**
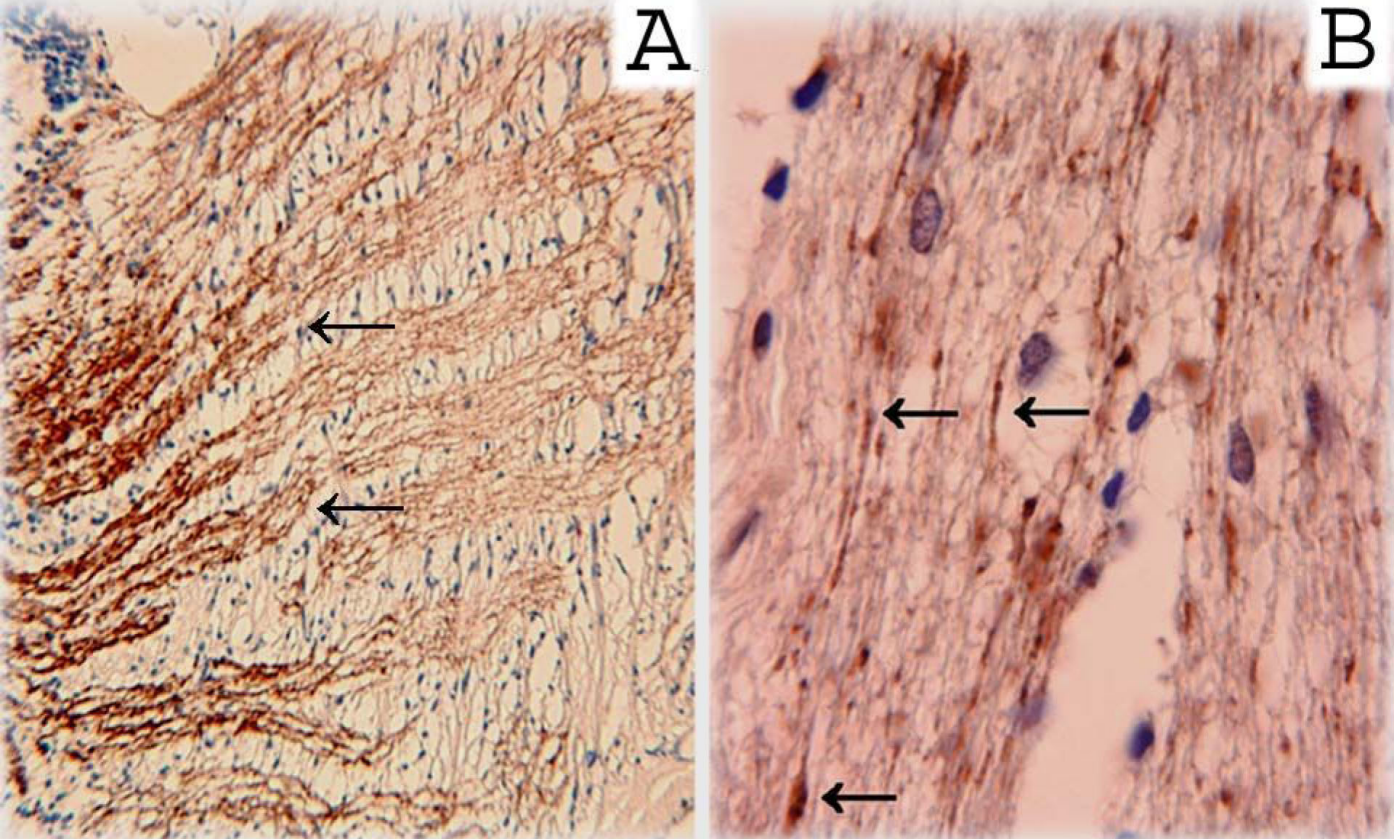
Immunohistochemical staining with a γ-synuclein antibody was used to examine a non-glaucomatous human optic nerve in the area immediately posterior to the lamina cribrosa (**A**) and the retrobulbar optic nerve from a patient with primary open-angle glaucoma (**B**). In both sections, γ-synuclein reactivity can be seen along presumptive retinal ganglion cell axon bundles. In section **B,** arrows show swollen axons and axons fragment immunopositive for γ-synuclein. These results confirm the presence of γ-synuclein in axons of RGC and its immunopathology in glaucoma.

Relative quantification was conducted in a two step qRT–PCR protocol using TaqMan starter kit reagents, Quantitative Real-Time PCR kits, and a 7300 series instrument from the manufacturer (Applied Biosystems). The following probes specific for rat mRNAs were used: Rn 00569821-m1 for α-synuclein, Rn00589963-m1 for β-synuclein, Rn00581652-m1 for γ-synuclein, and Rn00667869-m1 for β-actin. The latter was used as an endogenous control. Reverse transcription was carried by incubating the RNA samples with 50 U/μl of reverse transcriptase (RT; 10 min, 25 °C, 120 min, 37 °C) using High Capacity cDNA Reverse Transcription kit (Applied Biosystems) and following recommendations of the manufacturer. A minus RT control containing all the reaction components except the reverse transcriptase was included in all qRT–PCR experiments to test genomic DNA contamination. Amplification was performed using TaqMan Universal PCR master mix (Applied Biosystems) and the following conditions: 2 min at 50 °C (hold), 10 min at 95 °C (hold); 15 s at 95 °C, 45 cycles; 1 min 60 °C (hold). The samples were amplified in duplicate, and three independent isolations of RNA/cDNA were used. Control samples in which cDNA was not added did not give any amplification signals. Amplification products were confirmed by sequencing.

### Enzyme linked immunoassay of synuclein

Wells in each ELISA plate were coated with 1 µg/ml of γ-synuclein antibody E-20, sc-10698 (Santa Cruz Biotechnology, Inc., Santa Cruz, CA) in 50 µl of 50 mM sodium carbonate, pH 9.6 (coating solution) by an overnight incubation at 4 °C. The next morning the wells in the plate were washed three times with a 0.1% solution of PBS-Tween-20 and incubated with 200 µl/well of blocking solution (Super Block Blocking buffer in PBS; Pierce, Rockford, IL) for 30 min at 37 °C. Next, the plate was washed again with the PBS-Tween buffer. For the calibration curve, recombinant γ-synuclein [22] was used from 2.0 to 2000 pg/ml. In addition, a set of ten-fold dilutions of the RGC-5 or A7 cell extracts were added in the neighboring wells and incubated for 2–3 h at 37 °C. The wells were washed with PBS-Tween, then a secondary antibody, syn15–S (Alpha-Diagnostic, San Antonio, TX), was added at a 1:5,000 dilution and incubated for 1 h at 37 °C. The washing procedure with PBS-Tween was repeated, and an incubation with 1:5,000 antirabbit-HRP (Invitrogen) as a developing antibody solution was performed for 30 min at 37 °C. After washing, 100 µl/well of 3, 3′, 5, 5′ tetramethylbenzidine (TMB) substrate was added (ImmunoPure TMB substrate, Pierce, Rockford, IL). When blue color devoloped, 100 µl per well of 2N H_2_SO_4_ was added to stop the reaction, and the absorbance was measured at 450 nm.

## Results

We previously found that γ-synuclein is expressed in RGCs [23]. Here we explored whether γ-synuclein expression is RGC-specific. In sections of the human retina (Figure 1), γ-synuclein antibody stained the cytoplasm and processes of cells in the ganglion cell layer (GCL) that were positive for the RGC marker Brn-3a. All Brn-3a-positive nuclei were surrounded by γ-synuclein-positive cytoplasm. Cells not stained by either of these two markers (Figure 1) may represent displaced amacrine cells normally found in the GCL. Thus γ-synuclein appeared to demonstrate RGC-specific expression in human retina. The presence of a weak, punctate signal in the inner plexiform layer (IPL) in these retinal sections (Figure 1) may be explained by slight cross-reaction of γ-synuclein antibody with α-synuclein, which is localized in the IPL [23]. Thus in human retinal sections, γ-synuclein colocalizes with Brn-3a-positive RGCs.

We next considered whether γ-synuclein expression changed in retinal disease. In the retinas of patients with retinoblastoma, γ-synuclein immunoreactivity was reduced in the nerve fiber layer (NFL), but increased in the cell bodies of RGCs in the GCL (Figure 2). In a second retinoblastoma patient, we observed similar changes in the pattern of staining (data not shown). In three glaucoma patients, swollen axons and axonal fragments were also immunopositive for γ-synuclein (in Figure 3 a representative sample is shown).

In the optic nerve of a patient with glaucoma, normal RGC axons, swollen axons, and axonal fragments were immunopositive for γ-synuclein (Figure 3). Thus in example eyes with either retinoblastoma or glaucoma, γ-synuclein changed its pattern of subcellular expression, but appeared to maintain RGC expression and specificity.

**Figure 4:**
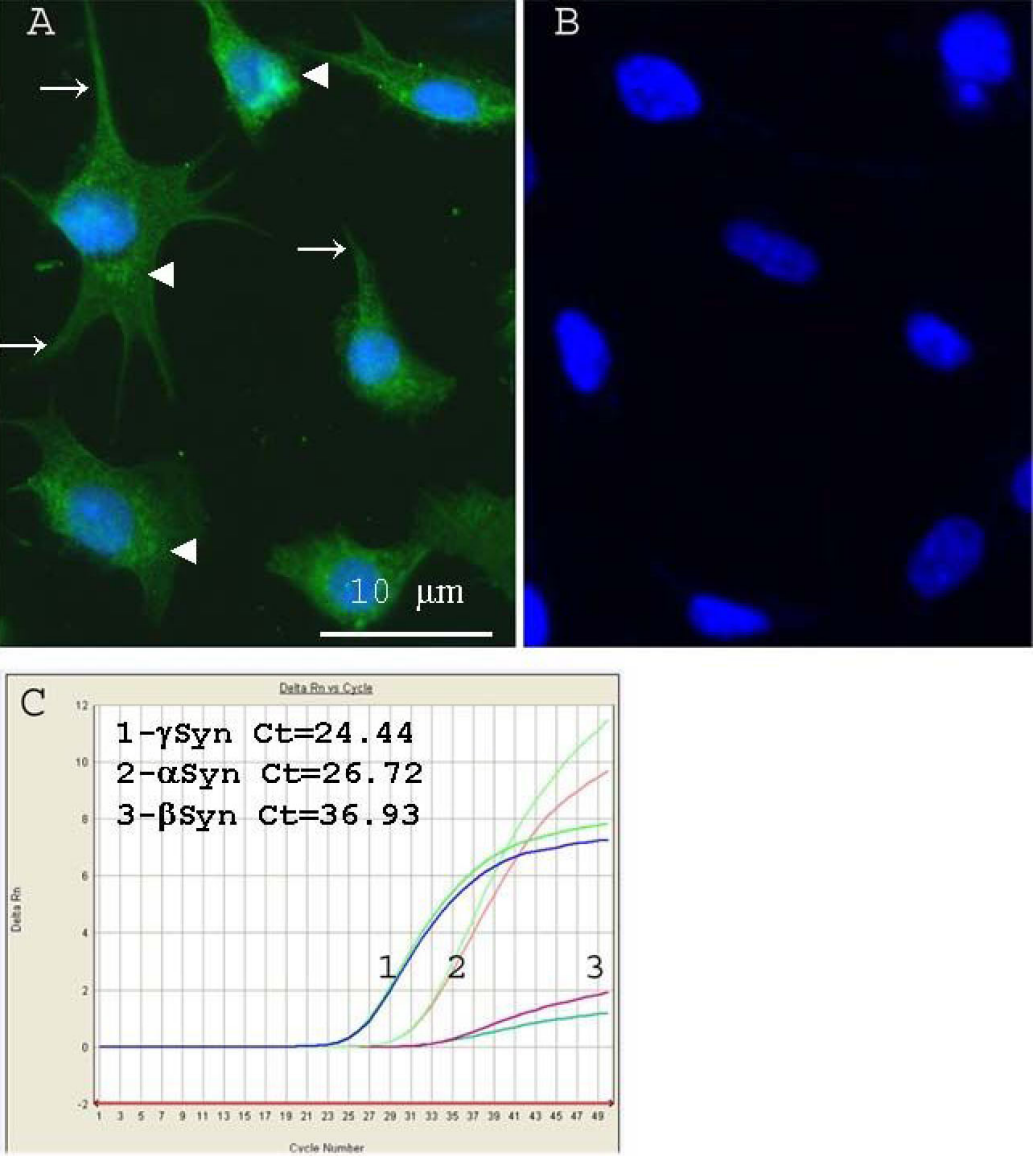
Retinal ganglion cell-5 (RGC-5) cell-line expression of γ-synuclein protein and mRNA were investigated using immunofluorescence and quantitative RT–PCR. **A:** Immunofluorescent staining of RGC-5 cultures reveals γ-synuclein staining (green) localized in the perinuclear area (arrowheads) and in cell processes (arrows). **B**: no fluorescence was detected in the absence of primary antibody.****Nuclei were counterstained with DAPI (blue). **C**:****qRT–PCR of endogenous synuclein-family transcripts in RGC-5 demonstrates that γ-Synuclein had the highest level of expression (Ct=24.44±0.3), followed by α-synuclein (Ct=26.72±0.3) and finally β-synuclein (Ct=36.93±0.4). Ct represents threshold cycles; a lower number reflects higher levels of initial mRNA. A representative duplicate curves are shown for three independent isolation. Ct differences were statistically significant (p<0.05).

We next analyzed the expression and localization of γ-synuclein in an immortalized RGC cell line, RGC-5. In RGC-5, γ-synuclein demonstrated a diffuse localization in the cytoplasm and in cell processes, with a typical punctate staining predominantly in the perinuclear area (Figure 4A,B). The nuclei of all these cells were stained with a polyclonal antibody against Brn-3a (not shown).

By quantitative RT–PCR, we analyzed γ-synuclein mRNA expression in RGC-5 cells. γ-Synuclein mRNA expression was more abundant compared to α- and β-synuclein levels with β-actin as an endogenous control (Figure 4C) . The threshold cycle (C_T_) was found to be 24.4±0.3 for γ-synuclein, 26.7±0.4 for α-synuclein and 36.9±0.4 for β-synuclein. Because γ-synuclein is localized in the optic nerve [13], we also considered the possibility that it is expressed by astrocytes. Although we did not generate primary cultures of optic nerve astrocytes, we were able to obtain the A7 astrocyte cell line. Using ELISA analysis, extracts of RGC-5 cells had significantly higher expression of γ-synuclein protein than A7 astrocyte cells (3.3 versus 0.8 µg/mg of total protein, p<0.01).

**Figure 5:**
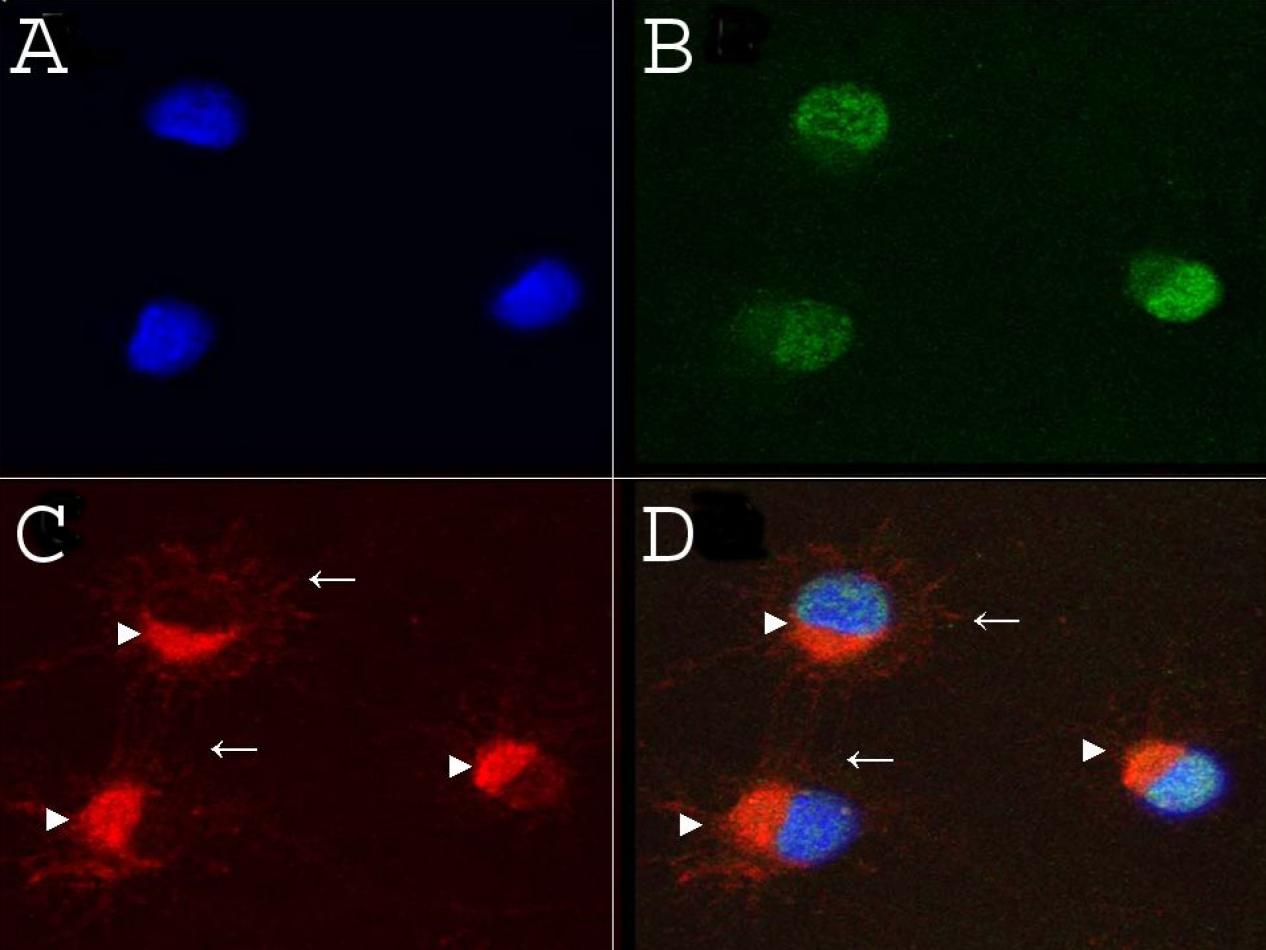
Localization of γ-synuclein and Brn-3a were examined in a primary culture of rat retinal ganglion cells (RGCs). **A**: DAPI (blue) counterstained the nuclei. **B**: Brn-3a (green) was localized to RGC nuclei. **C**: γ-synuclein (red) was localized throughout the cytoplasm (arrowheads) and processes. A merged image of **A, B**, and **C** is shown in **D**. Thus, in primary culture of RGCs, γ-synuclein is localized in the cytoplasm and processes, while Brn-3a in the nuclei.

**Figure 6:**
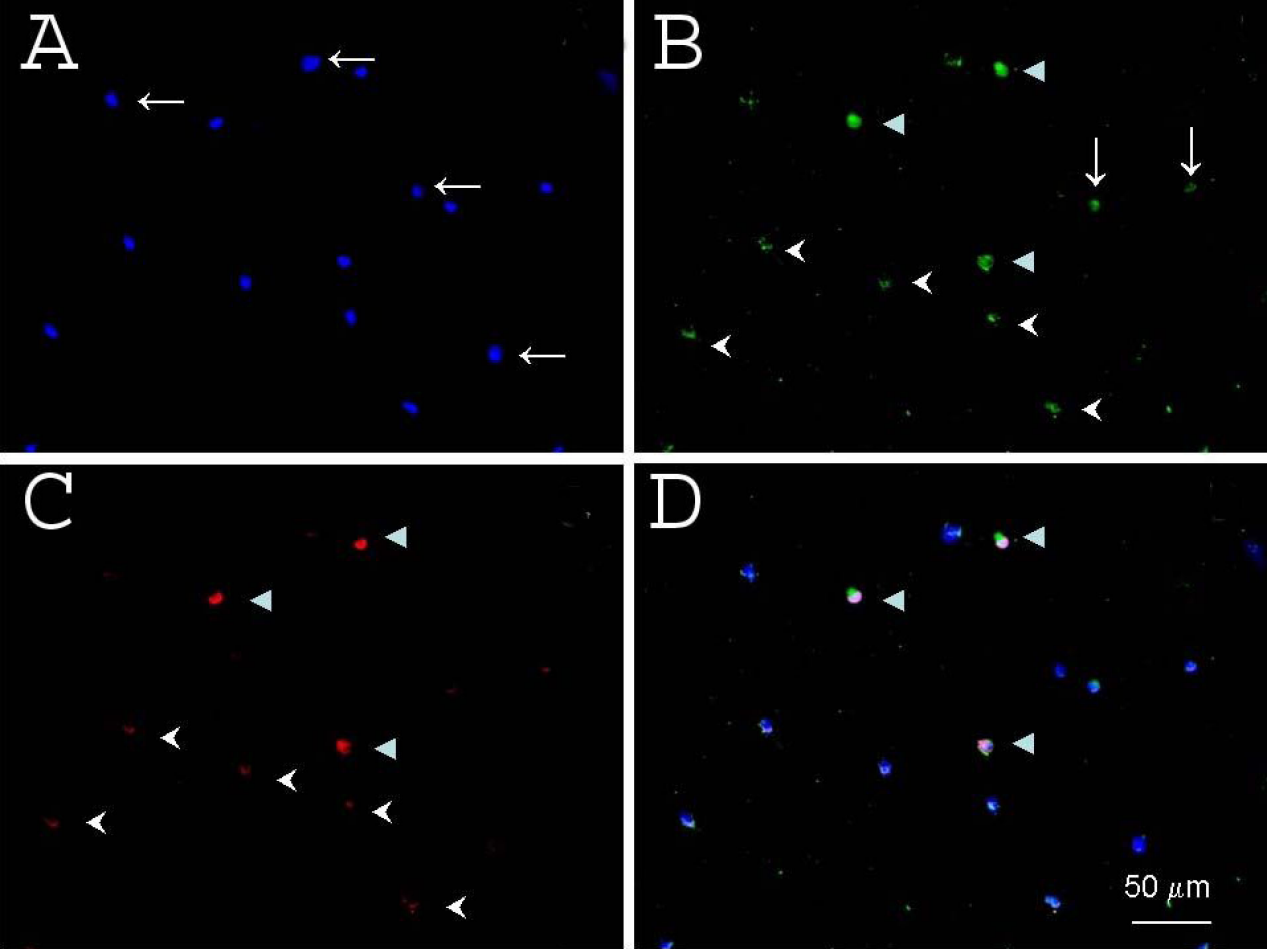
γ-Synuclein specificity was examined using immunofluorescent staining of rat retinal cells. **A**: DAPI (blue) counterstained the nuclei. **B**: Brn-3a (green) was localized to RGC nuclei. **C**: γ-synuclein (red) was localized throughout the cytoplasm (arrowheads) and processes (arrows). A merged image of **A**, **B**, and **C** is shown in **D**. Many cells demonstrated neither Brn-3a nor γ-synuclein reactivity (horizontal arrows in **A**). A subset of presumptive retinal ganglion cells demonstrated intensive staining by both γ-synuclein and Brn-3a (full arrowheads in **B**, **C**, and **D**), while many other cells showed only weak staining by both γ-synuclein and Brn-3a (arrowheads in **B** and **C**). A few cells were stained by γ-synuclein, but not by Brn-3a (vertical arrows in **B**).

**Figure 7:**
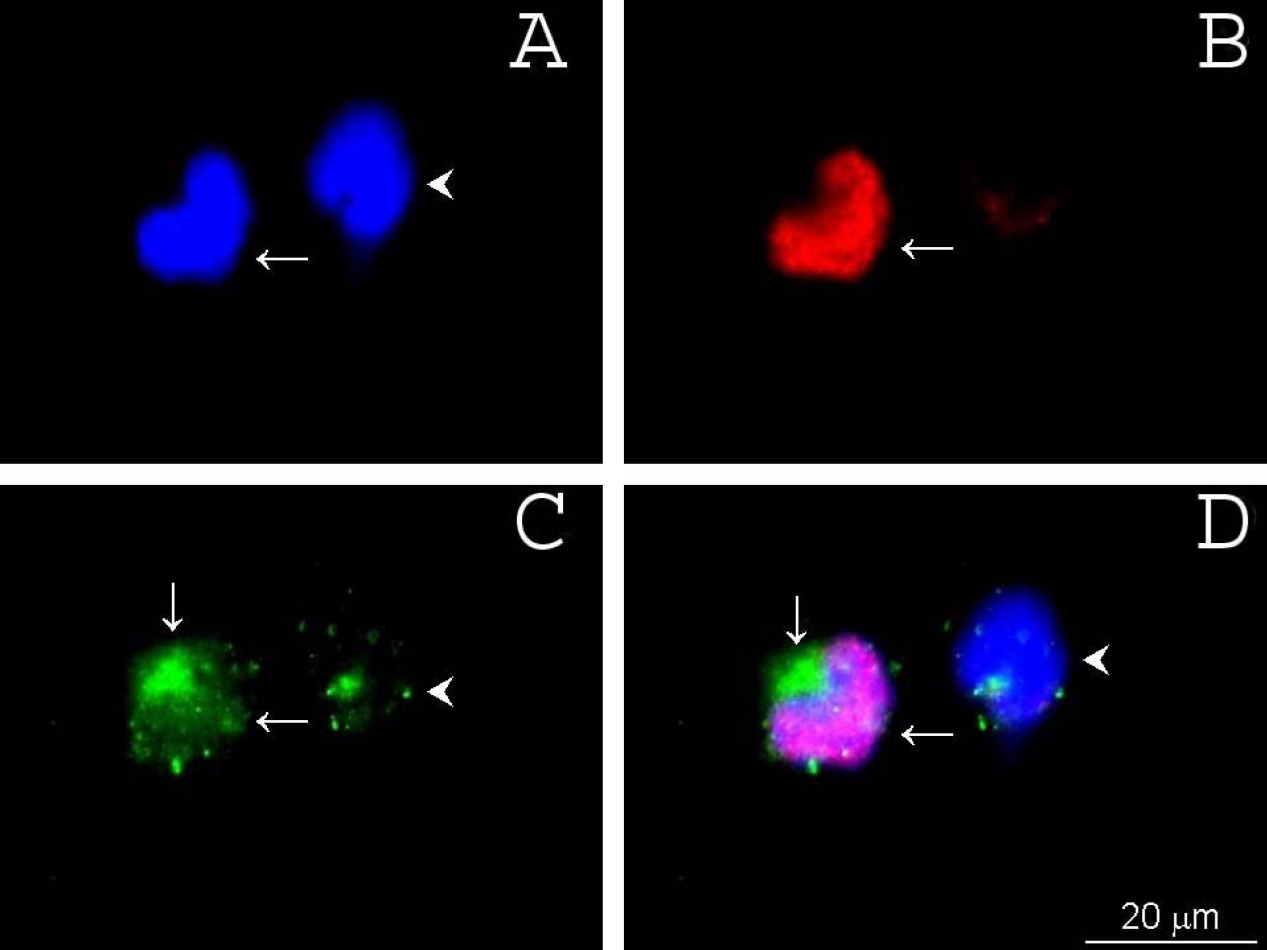
γ-Synuclein subcellular localization was examined using immunofluorescent staining of rat retinal cells. **A**: DAPI (blue) counterstained the nuclei. **B**: Brn-3a (red) was localized to RGC nuclei. **C**: γ-synuclein (green) was co-expressed by the Brn-3a expressing cell (arrows) but only weakly by a non-Brn-3a-expressing cell (arrowhead). A merged image of **A**, **B**, and **C** is shown in **D**. Immunofluorescent staining of retinal cell suspension. **A**: DAPI, **B**: Brn-3a, **C**: γ-synuclein, **D**: merged images. Horizontal arrows show a cell immunopositive for both Brn-3a and γ-synuclein. Arrowhead (**C** and **D**) points to a cell not stained by Brn-3a antibody that is stained by a γ-synuclein antibody. Vertical arrow (**C** and **D**)****points to the localization of γ-synuclein in the perinuclear area. Thus, brighter and broader cytoplasmic staining of γ-synuclein overlapped well with Brn-3a staining.

We next used cultures of rat retinal cells and rat RGCs purified by immunopanning to consider whether γ-synuclein was specific for RGCs. In primary cultures of purified rat RGCs, γ-synuclein was again localized in the cytoplasm, while Brn-3a was expressed in the nuclei (Figure 5). The asymmetry of the staining was mainly from the asymmetry of the cytoplasm. When retinal suspensions were immunostained with antibodies to γ-synuclein and Brn-3a, we identified four types of cells (Figure 6). Most abundant were cells not stained by either γ-synuclein or Brn-3a antibodies, as expected since RGCs make up a small fraction of the neurons in the retina. About 1% of retinal cells were marked by intense staining by both antibodies. Finally, there were retinal cells weakly double-positive, and rare γ-synuclein-positive/Brn3a-negative cells (Figure 6). We did not determine whether this last group represented a Brn-3a-negative subtype of RGC, or a different retinal cell type. We noted at higher magnification that Brn-3a-positive and Brn-3a-negative cells differed in their γ-synuclein immunoreactivity (Figure 7). Brn-3a positive cells were extensively stained throughout the cytoplasm by the γ-synuclein antibody, and more strongly in the perinuclear area where we observed bright, punctate reactivity. In contrast, Brn-3a negative cells demonstrated only a weak dot-like staining in a small part of the cell. In all cases, Brn-3a staining overlapped with DAPI nuclear staining (Figure 7). Thus, taken together, these data demonstrate that brighter and broader cytoplasmic staining of γ-synuclein overlapped well with Brn-3a staining, and that γ-synuclein appears to be an RGC-specific marker.

## Discussion

Here we show that γ-synuclein is a specific marker of RGCs, and that its expression and localization are altered in ocular diseases. We have demonstrated similar results with two different γ-synuclein antibodies in this study, and a third in a previous study [23], and have found similar patterns of RGC specificity in human and rodent retinas. The identification of specific molecular markers for RGCs, which may have more than a dozen subtypes [7,24,25], has been difficult. Previously, Thy1–1 and Brn-3 family proteins have been described as RGC markers [9-12,26]. However, their expression is developmentally regulated and Thy1 expression is not limited to RGCs, but is also weakly expressed by a subset of cells in the inner nuclear layer. In contrast, γ-synuclein mRNA and protein are expressed in neurons from the earliest stages of axonal outgrowth and are maintained at a high level throughout life [27]. γ-Synuclein and other such markers may be useful to investigate normal functions of RGCs and the mechanisms underlying their death [16,28].

Synucleins are small natively unfolded proteins implicated in neurodegenerative diseases and some forms of cancer [27,29-31]. We and others previously found that three members of the synuclein family were differentially expressed in the retina [23,32]. α- and β-Synucleins are expressed mainly in the inner plexiform layer (IPL), inner nuclear layer, and in photoreceptor cells, whereas here we find that γ-synuclein is expressed by RGCs in the ganglion cell layer and in their axons in the nerve fiber layer (NFL). Here we found that, in the retinas of retinoblastoma patients, γ-synuclein expression was reduced in the NFL and increased in the cell bodies of subsets of RGCs. In patients with primary open-angle glaucoma, the bundles of axons and axon swellings remained immunopositive for γ-synuclein. The role of this protein in retinal cancers and retinal degenerative diseases remains to be studied, but these data suggest that γ-synuclein’s role in other disease processes [30,31] may be mirrored in the eye. For example, synucleins bind to vesicles through their N-terminal repeat region and are involved in axonal transport [33]. The accumulation of γ-synuclein aggregates we observed in a glaucomatous optic nerve might be a result of the disruption of γ-synuclein transport in axons of RGCs, consistent with the decrease in γ-synuclein expression and the axonal transport impairment in the DBA/2J mouse model of glaucoma [34]. γ-Synuclein could also play a causative role in axonal transport deficits through its ability to modulate neurofilament network integrity [29] or through its ability to upregulate matrix metalloproteinase 9, an enzyme modulating extracellular matrix [22] that plays a role in retinal ganglion cell death in animal models [35].

In this study our antibodies did not distinguish expression of differentially modified γ-synuclein proteins in RGCs and RGC axons. In a previous study, it was demonstrated that all three synucleins are substrates of G-protein coupled receptor kinases [36] which phosphorylate Ser-129 in α-synuclein and the corresponding Ser-124 in γ-synuclein. Other posttranslational modifications have been described for synucleins, including sumoylation, O-glycosylation, tyrosine nitrosylation among others [37,38]. It would be interesting to determine whether γ-synuclein is differentially modified during development or in disease.

Taken together, these data demonstrate that γ-synuclein is highly expressed in RGCs, and that this high level of expression is largely RGC-specific. The use of a γ-synuclein promoter [39] for specific delivery of siRNA or fluorescent markers to RGCs might be useful for future translational approaches. We propose that this marker may become an important tool for the future investigation of normal physiology of RGCs and the mechanisms underlying their death, and is itself a strong candidate for playing a role in the pathophysiology of RGC death in glaucoma and other degenerative diseases.
